# Anti-Inflammatory Properties In Vitro and Hypoglycaemic Effects of Phenolics from Cultivated Fruit Body of *Phellinus baumii* in Type 2 Diabetic Mice

**DOI:** 10.3390/molecules26082285

**Published:** 2021-04-15

**Authors:** Kai Yang, Su Zhang, Yan Geng, Baoming Tian, Ming Cai, Rongfa Guan, Yougui Li, Bangwei Ye, Peilong Sun

**Affiliations:** 1 College of Food Science and Technology, Zhejiang University of Technology, Hangzhou 310014, China; yangkai@zjut.edu.cn (K.Y.); zhangsu19940328@163.com (S.Z.); wlgengyan@163.com (Y.G.); caiming@zjut.edu.cn (M.C.); guanrongfa@zjut.edu.cn (R.G.); sun_pl@zjut.edu.cn (P.S.); 2 Sericultural Research Institute, Zhejiang Academy of Agricultural Sciences, Hangzhou 310021, China; liyougui3@163.com; 3 Zhejiang WisePlus Health Technology Co., Ltd., Lishui 323006, China; bondesse@aliyun.com

**Keywords:** *Phellinus baumii*, phenolics, type 2 diabetes mellitus, anti-inflammatory properties, hypoglycemic effects

## Abstract

Dietary intervention in type 2 diabetes mellitus (T2DM) is a hotspot in international research because of potential threats to human health. *Phellinus baumii*, a wild fungus traditionally used as a food and medicine source, is now cultivated in certain East Asian countries, and is rich in polyphenols, which are effective anti-inflammatory ingredients useful in treatment of T2DM, with fewer side effects than drugs. To examine the hypoglycaemic effects of *Phellinus baumii* phenolics (PPE), the metabolite profiles of T2DM mice induced by streptozotocin after PPE intervention were systematically analyzed. Here, 10 normal mice were given normal saline as control group, and 50 model mice were randomly assigned to five groups and daily intragastric administrated with saline, metformin (100 mg/kg), and PPE (50, 100, 150 mg/kg of body weight), for 60 days. The pro-inflammatory factor contents of lipopolysaccharide stimulation of RAW 264.7 cells were decreased in a dose-dependent manner after PPE treatment, we propose that PPE could exert anti-inflammatory properties. PPE could also effectively reduce blood glucose levels, increased insulin sensitivity, and improved other glucolipid metabolism. Q-PCR results suggested that the hypoglycemic effects of PPE might be through activating IRS1/PI3K/AKT pathway in diabetic mice. These results suggest that PPE has strong potential as dietary components in the prevention or management of T2DM.

## 1. Introduction

Type 2 diabetes mellitus (T2DM) is a very common disease that affects citizens in both developed and developing countries. The prevalence of T2DM in adult has risen from approximately 150 million in 2000 to about 450 million in 2019, and is predicted to rise up to 700 million in 2045 [1]. Genetics and lifestyle habits can exert a predisposing influence on T2DM incidence, and there are differences among people of different racial and/or ethnic backgrounds, with varying degrees of insulin resistance (IR) [2]. The contributions of environmental factors (e.g., sedentary lifestyle, high-calorie diet, obesity) are considered to be greater than that of genetic factors [3]. T2DM accounts for 90–95% of those with diabetes. Therefore, different intervention strategies to prevent T2DM are key to controlling the high rate of diabetes growth. T2DM can lead to the chronic injury and even dysfunction of liver, kidneys, pancreas, nerves and other tissues [4]. In addition, T2DM sufferers are usually accompanied by a series of abnormal symptoms of their bodies, e.g., lipid and protein metabolism disorder, oxidative stress, subclinical inflammation and vascular sclerosis. Diabetes and its concomitant diseases pose serious threats to the quality of human life, and the World Health Organization (WHO) has ranked it as the third largest non-genetic disease after cancer and cardiovascular disease. Though a number of synthetic medicines are available for managing T2DM, their long-term use is associated with various adverse effects [5], e.g., lactic acid poisoning, hepatotoxicity and gastrointestinal dysfunction, etc. [6]. Diet and exercise play a very important role in preventing and treating T2DM, e.g., a hierarchical and accurate dietary management of T2DM contributes to optimizing dietary recommendations in terms of carbohydrates, fats and dietary fiber [1]. It has shifted the research towards natural products which are considered to be comparatively safe. Edible mushrooms are rich in natural compounds, e.g., fibers, polysaccharides and phenolics, known for providing antidiabetic, anti-inflammatory and antihyperlipidemic effects from ancient times, and are a kind of typical organisms with potential anti-diabetes effect [5]. At present, various natural anti-diabetic active ingredients have been paid more and more attention including polyphenols, polysaccharides, essential oils, trace minerals and organic acids, etc., derived from edible fungi with fewer side effects than drugs.

*Phellinus baumii* (*P. baumii*), known as “Sang Huang” in China, has traditionally been used as a healthy food or folk tonic in East Asia because of its multiple physiological functions, e.g., reducing blood lipid levels, anti-tumor, anti-influenza, anti-oxidation capacities, DNA damage-protecting, immune-stimulating and anti-diabetic activities [7,8,9,10]. The nutritional value and functional value of *P. baumii* have attracted worldwide attention, and people began to study its nutritional function in depth. The ethanol and ethyl acetate extracts of *P. baumii* have also been found to show hypoglycemic effects in streptozotocin-induced diabetic mice [11]. Members of the genera *Phellinus* are well-known edible and medicinal fungi and some of them have been used to treat diabetes with few adverse effects. According to previous studies, *Phellinus fungus* contains a variety of yellow polyphenolic compounds, e.g., hispidin, which has shown significant biological effects and has been used to treat diabetes, bacterial and viral infections, ulcers and, cancer [12]. Few side effects of these mushrooms have been reported, suggesting that these mushrooms are considered safe by current studies. In modern medical research, polysaccharides [8] and polyphenols (including flavonoids) [9,13,14] as constituents of *Phellinus fungus*, they have been reported as the main bioactive constituents with a wide range of health effects and biological activity. The phenolics separated from the fruit body of wild *Phellinus* exhibited hypoglycemic effects, as well as antioxidant and neuroprotector influence [15]; at present, the fruiting body of *Phellinus baumii* has been cultivated on a large scale. Our previous studies showed that extracts, especially of ethyl acetate fraction (PPE) consisting mainly of 6 phenolic compounds, from cultivated fruit body of *Phellinus baumii* demonstrated a potentially hypoglycemic effect in vitro [14]. Furthermore, it is still unknown whether PPE also contribute hypoglycemic biological activities in vivo. The aim of this study was to explore the effect of PPE on the reversal of IR, especially on the regulation of glucose metabolism disorders in streptozotocin-induced T2DM mice.

## 2. Materials and Methods

### 2.1. Preparation of PPE

**Figure 1:**
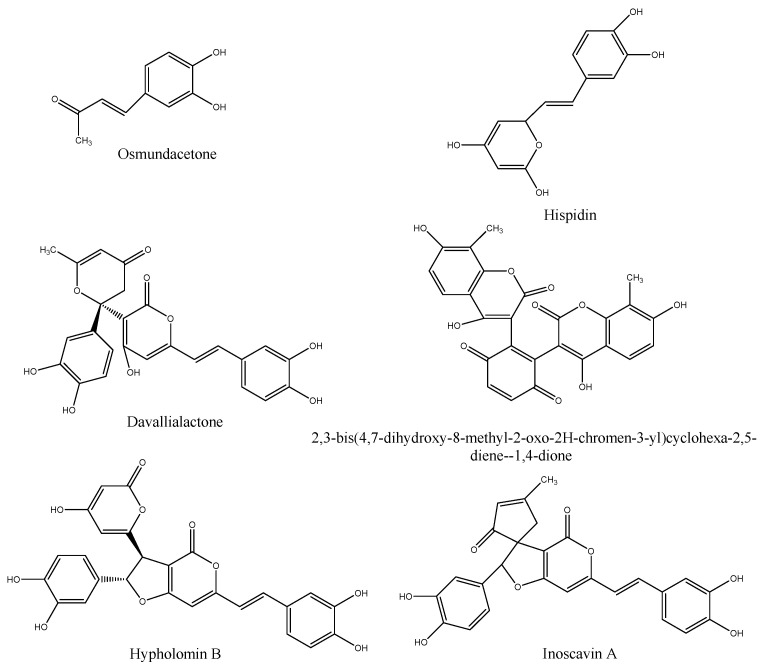
Chemical structures of 6 major compounds identified from PPE.

Fruiting bodies of *P. baumii* were cultivated by the Sericultural Research Institute, Zhejiang Academy of Agricultural Sciences (Hangzhou, China), and the species was identified by Professor Lizhong Fu, Zhejiang Chinese Medicine University (Hangzhou, China). According to our previous methods, PPE was prepared by ethyl acetate extraction [14]. The structures of the main phenolic compounds in PPE were shown and introduced with more details in our previous research as described below [14]. The phenolic purity of PPE was 79.45 ± 0.48% by a Folin-phenol method against the dry weight of PPE. As presented in our previous research, six major polyphenolic compounds in PPE were tentatively identified by liquid chromatography−mass spectrometry (LC-MS). The main phenolic compounds in PPE were identified as osmundacetone, hispidin, davallialactone,2,3-bis(4,7-dihydroxy-8-methyl-2-oxo-2*H*-chromen-3-yl)cyclohexa-2,5-diene-1,4-dione, hypholomin B, and inoscavin A, and the relative area of each peak was 1.09, 2.96, 13.72, 10.70, 34.66, and 6.32%, respectively (Figure 1). As seen from their structure (Figure 1), most of them contained the major part of hispidin.

### 2.2. Measurement of Anti-Inflammatory Activity In Vitro

The RAW 264.7 cell line was provided by the Beijing Institutes of Life Science, Chinese Academy of Sciences. The cell viability assay was carried out, as previously reported [16]. In brief, 264.7 protocells were inoculated in a 96-well plate (1.0 × 10^5^ cells/well) with RPMI 1640 and 10% FBS for 12 h in a wet environment with 5% CO_2_ at 37 °C. The sample solutions of different concentrations (12.5, 25, 50, 100 μg/mL PPE) next were pretreated for 2 h, followed by 10 ng/mL lipopolysaccharide (LPS) for 24 h.

The medium was sucked out by pipette, and 10 μL of 5 mg/mL MTT solution was added to each well and incubated at 37 °C for 4 h, supplied 100 μL DMSO after blow-drying the supernatant, then read and the optical density value at 490 nm of each well calculated. The levels of TNF-α and IL-1β were performed by a commercial enzyme-linked immunosorbent assay (ELISA) kit. The PPE free sample solution and indomethacin (positive control, Sigma-Aldrich, St. Louis, MO, USA) were treated in the same batch with LPS (10 ng/mL). The supernatant was collected after centrifugation and the values of the aforementioned factors were determined.

### 2.3. Animal Breeding Conditions and Treatments

A total of 100 ICR male mice (8-week-old, 18–22 g) were purchased from Shanghai Lingchang Biotechnology Co., Ltd. (Shanghai, China) and fed in a standard animal room (12 h light, 12 h dark cycle, 22 °C ± 1 °C, relative humidity of 55 ± 5%). After adaptive feeding for one week, fifteen mice were randomly selected as normal control group. T2DM was induced as described in a previous study [17]. The remaining 85 mice were induced by a high-fat diet for 30 days, fasted for 16 h, and then induced T2DM by intraperitoneal injection of streptozotocin (STZ). That is, on the 1st and 4th days, STZ (30 mg/kg) was intraperitoneally injected, and blood glucose was assessed in the tail vein 72 h after injection. A total of 50 mice with blood glucose level ≥ 11.1 mmol/L were selected as T2DM model mice. 50 model mice were randomly divided into the following 5 experimental groups (10 mice in each group). The normal control group (NC) in group I was fed the conventional diet with normal saline intragastrically; group II was fed with the high-fat diet, with saline as the model control (MC); the model control group (MC) in group II were fed the HFD with normal saline intragastrically; positive control group (Met) was fed with the HFD and incorporated with metformin (100 mg/kg/bw); group IV (L), V (M) and VI (H) were fed the HFD diet given PPE (50, 100, and 150 mg/kg/bw, respectively) intragastrically; Saline, metformin and PPE were given daily via oral gavage for 60 days. The conventional and high-fat diet was purchased from Jiangsu synergetic pharmaceutical bioengineering Co., Ltd. (Nanjing, China). The NC group and MC group were given the same volume of normal saline. The body weight and fasting glucose level after fasting 12 h of each mouse were recorded every 10 days. After fasting for 12 h, anesthetized mice were given eyeball and blood samples were centrifuged at 4000× *g* for 10 min to obtain serum, which was stored at −80°C for further analysis. The program for animal experiments was approved by the Animal Ethics and Experimental Committee of Jiangsu KeyGEN BioTECH Corp., Ltd. (Nanjing, China).

### 2.4. Determination of Serum and Tissue Biochemical Index in Mice

The commercial assay kits for epinephrine (EP, H208), high-density lipoprotein cholesterol (HDL-C, A112-1-1), insulin (INS, H203-1-2), low-density lipoprotein cholesterol (LDL-C, A113-2-1), glycosylated hemoglobin (HbA1c, H464-1), glucagon (GCG, H183), and serum triglyceride (TG, A110-1-1) were determined by the instructions of commercial detection kits from Nanjing Jiancheng Bioengineering Institute (Nanjing, China).

### 2.5. Oral Glucose Tolerance Test

Oral glucose tolerance test (OGTT) was performed within one week before the end of treatment. The mice fasted for 16 h with water and were treated with a glucose meter (Roche, Basel, Switzerland) to determine fasting blood glucose.A 2 g/kg/bw glucose solution was rapidly gavaged, and blood glucose values were measured at 30, 60, 90 and 120 min after gavage [18]. The area under the OGTT curve (AUC) value was calculated after the glucose value was taken from 0 to 120 min by gavage of glucose.Insulin resistance index (HOMA-IR) was calculated as follows [19]:

$$\mathrm{HOMA}\;-\;\mathrm{IR}\;=\left(\mathrm{FPG}\;\times \;\mathrm{FINS}\right)/22.5$$

In the formula, FPG and FINS represent fasting blood glucose and fasting insulin levels, respectively.

### 2.6. Quantitative Real-Time PCR

Total RNA was extracted from liver tissues using TRNzol Universal Reagent (Tiangen Biotech Co. Ltd., Beijing, China). After that, RNA was reverse-transcribed into cDNA by reverse transcription of 1 μg of total RNA (Tiangen Biotech). PCR amplification was performed according to the method as described previously [20]. The primers for Quantitative real-time PCR (Q-PCR) primers of PI3K, AKT, IRS1, and β-actin were synthesized by Shanghai Sangon Biotech Co. (Shanghai, China) and is provided in Table S1. The results were expressed as relative mRNA expression compared to normal mice by 2^−ΔΔCt^ method using β-actin as internal reference.

### 2.7. Hematoxylin and Eosin Staining

After the mice were dissected, the liver tissue was fixed with 4% formaldehyde fixation solution for 24 h, embedded and sected with paraffin wax, and then treated with sappanwood Hematoxylin-eosin staining (H&E) was observed based on our previous research [21]. The study was observed and photographed under an inverted optical microscope (Eclipse Ti2 microscope, Nikon, Tokyo, Japan, 200×).

### 2.8. Statistical Analysis

Results were expressed as the mean ± standard deviation (SD). Intergroup comparisons were performed by one-way analysis of variance (ANOVA) using SPSS 19.0 software program (SPSS Inc., Chicago, IL, USA) with LSD’s test. A *p* value of less than 0.05 or 0.01 is significant and very significant, respectively.

## 3. Results and Discussion

### 3.1. Anti-Inflammatory Properties of PPE

**Figure 2:**
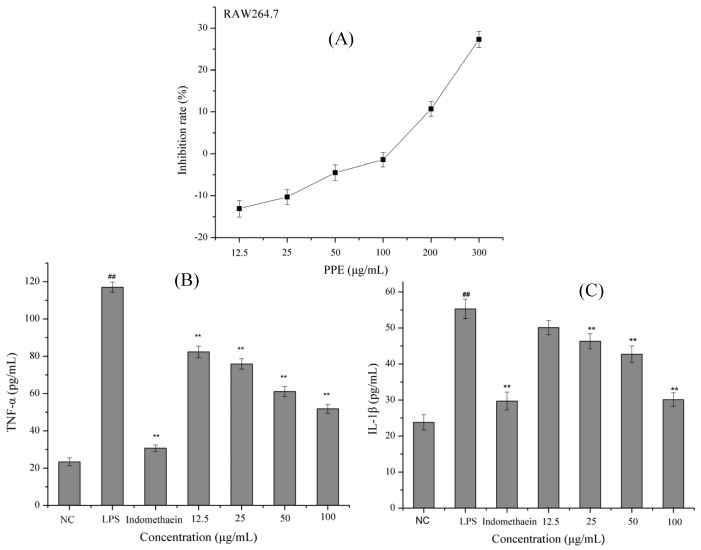
Anti-inflammatory properties of PPE. (**A**) Inhibition rate of different concentrations of PPE on RAW264.7 cells; The inhibitor effect of samples on the LPS-induced TNF-α (**B**) and IL-1β (**C**) production. ^##^ *p* < 0.01 vs. NC; ** *p* < 0.01 vs. MC.

As shown in Figure 2A, the growth of RAW264.7 cells was not inhibited when PPE concentration was below 100 μg/mL, but it was severely inhibited when PPE concentration increased from 100 to 300 μg/mL, suggesting that PPE had little cytotoxic effects on RAW 264.7 cells at the levels of 12.5–100 μg/mL, but PPE showed obvious cytotoxicity at above 100 μg/mL. Based on the results, 12.5, 25, 50 and 100 μg/mL PPE were selected for next anti-inflammatory activity study. According to previous studies that the various bioactive components of *P. baumii* could exert immune enhancing effects on anti-inflammatory properties of *P. baumii* in LPS-stimulated RAW 264.7 cells [7]. In vitro studies showed that *P. baumii* extract (0.01–10 mg/mL) had no effect on spleen cell viability in healthy normal mice. Acute oral toxicity studies have reported few adverse or toxic effects of *Phellinus* species at 5 g/kg/bw [7].

As an important inflammatory factor, TNF-α is mainly expressed by macrophages and is closely related to the formation of many diseases [22]. For example, TNF-α is highly associated with the formation of T2DM, and the expression level of TNF-α is much increased in T2DM compared with that in the normal person [23]. IL-1β is an inflammatory cytokine and is considered to be the most critical factor contributing to T2DM [24]. Excessive production of IL-1β directly leads to apoptosis and dysfunction of β cells, and impairing insulin secretion sequentially [25]. Therefore, IL-1β may be a target for adjuvant therapy in T2DM. Moreover, IR phenomenon occurs in the body, the level of proinflammatory factors in peripheral blood increases, and the content of anti-inflammatory factors decreases [26]. In the early stages of inflammatory response, it is strongly initiated and mediated by inflammatory mediators such as prostaglandin E2 (PGE2), pro-inflammatory cytokines (TNF-α, IL-1β, and IL-6) [27]. In turn, these increased inflammatory cytokines further stimulate the biosynthesis and release of other inflammatory cytokines, ultimately leading to severe clinical symptoms of immune disorders and pain, ect [28]. Moreover, increased TNF-αlevels were linked to IR and T2DM, and decrease insulin sensitivity by affecting insulin receptor phosphorylation [29]. Furthermore, IL-1β-mediated autoinflammatory processes also lead to β-cell death [29]. Therefore, we used ELISA kit to detect the levels of TNF-α and IL-1β in LPS-stimulated RAW 264.7 cells to explore the effects of PPE on inflammatory regulators and the mechanism of intervention of PPE on IR. The effects of PPE on TNF-α are shown in Figure 2B,C. LPS significantly increased the inflammatory factor TNF-α and IL-1β in RAW264.7, which was inhibited by indomethacin administration. In anti-inflammatory experiments, indomethacin is usually used as a positive control, and the data also confirmed that TNF-α and IL-1β were inhibited by indomethacin in RAW264.7 after LPS treatment. When RAW264.7 was treated with different concentration PPE (12.5–100 μg/mL), TNF-α and IL-1β were reduced in a dose-dependent manner compared to those in the NC group (*p* < 0.01). The accumulating results suggested that PPE reduced the risk of T2DM by decreasing the contents of the inflammatory cytokines e.g., TNF-α and IL-1β. In healthy conditions, inflammation is thought to be a short-term or acute process that contributes to in tissue repair. while, if the inflammation persists for a long time, it can lead to damaging effects, such as IR [26].

### 3.2. Effect of PPE on the Body Weight Gain and Blood Glucose Level

The effects of PPE on body weight in mice are summarized in Table 1. The weight of mice in the MC group were increased compared with that in the NC group at different periods, which was due to the different diet given to mice during the model building. Compared with the MC group, the body weights were higher than that in the metformin-treated (*p* < 0.05), 50 (*p* < 0.01), 150 (*p* < 0.01) mg/kg PPE-treated groups at the 40th day. In comparison with the MC group, oral treatment with metformin significantly decreased body weight when mice were fed for 30 days until the end (*p* < 0.05), as expected, PPE significantly reduced weight gain in a dose-dependent manner after feeding for 40 days (*p* < 0.05). The results showed that all the low (L), medium (M) and high (H) dose of PPE had a significant effect on weight gain in diabetic mice compared with the MC group in a dose-dependent manner after of intervention of 40 days.The weight loss effect of each dose PPE was consistent with that of metformin group after 60 days compared to the MC group. T2DM mouse model was successfully established by weight detection. In order to evaluate the effect of intragastric PPE on T2DM model mice, we further examined the changes of a series of metabolic parameters in T2DM mice treated with different dose PPE.

### 3.3. Dynamic Changes of Blood Glucose during PPE Treatment in Mice

**Figure 3:**
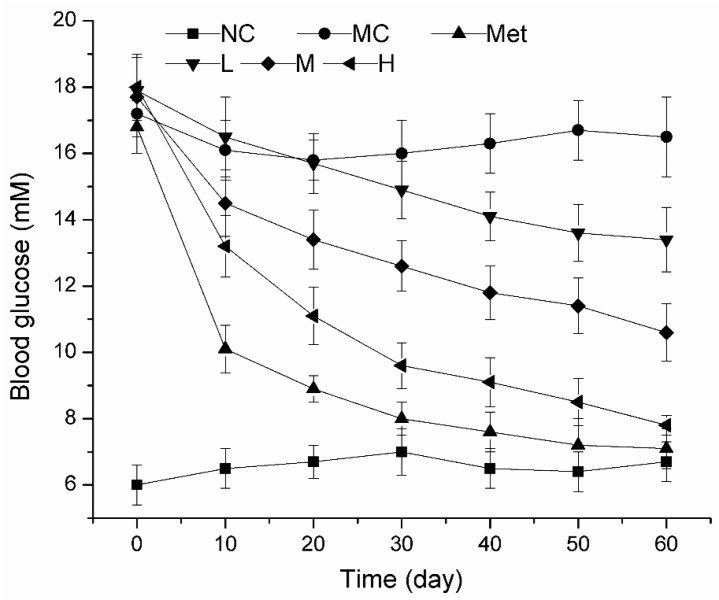
Effect of PPE on blood glucose in mice NC, normal control group; MC, model control group; Met, positive control group; L, M, and H groups administrated with 50, 100, and 150 mg PPE/kg/bw, respectively.

Blood glucose level is the most intuitive manifestation of diabetes. STZ can inhibit the activity of key enzymes required for gluconeogenesis in glucose metabolism, and it is commonly used to build a diabetes model in vivo. When the blood glucose level of STZ-treated mice is above 11.1 mM, the diabetic mouse model was considered to be built successfully. As shown in Figure 3, the blood glucose level of the MC group (15.2 ± 0.9 mM) was significantly increased compared with the NC group (6.0 ± 0.6 mM), indicating that the mice were insulin resistant after STZ injection and the model was successfully constructed. After 100 mg/kg/bw metformin was administered daily, the blood glucose level of model mice decreased significantly (*p* < 0.01). When the model group mice were given PPE administration for a period of time, the blood glucose decreased in a dose-dependent manner. In particular, after 60 days of PPE administration at a dose of 150 mg/kg/bw, the blood glucose of the mice could be reduced to 7.8 ± 0.3 mM, this effect was similar to metformin. Thus, PPE can reduce the risk of hyperglycemia by improving blood glucose levels in T2DM mice.

### 3.4. Effects of PPE on OGTT in Mice

**Figure 4:**
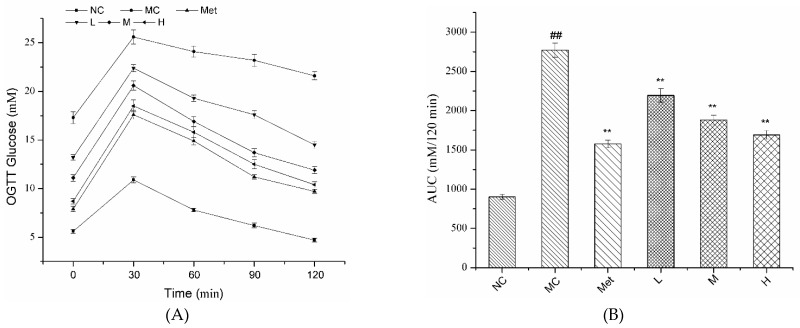
Effect of PPE on oral glucose tolerance test (OGTT) in mice. NC, normal control group; MC, model control group; Met, positive control group; L, M, and H groups administrated with 50, 100, and 150 mg PPE/kg/bw, respectively. (**A**) OGTT; (**B**) area under curve (AUC) analyses for OGTT; ^##^ *p* < 0.01 vs. NC, ** *p <* 0.01 vs. MC.

The changes of OGTT glucose in each group within 120 min are shown in Figure 4A. Blood glucose in all groups exhibited an upward trend in 0–30 min and a downward trend in 30–120 min after glucose administration. Both PPE and metformin treatment groups effectively alleviated abnormal glucose tolerance in diabetic mice with a dose-dependent effect. As shown in Figure 4B, the AUC of OGTT within STZ injected mice was significantly increased compared with the NC group (*p* < 0.01). Metformin significantly reduced AUC of OGTT in diabetic mice. After 60 days of PPE administration, the treated mice had lower AUC of OGTT compared with type 2 diabetic mice, and the decreased AUC values were more prominent with increased PPE concentration from 50 to 150 mg/kg/bw. The results demonstrated that PPE alleviated glucose tolerance and exerted a positive effect on the treatment of diabetes.

### 3.5. Effects of PPE on Insulin and Insulin Resistance Index in Mice

**Figure 5:**
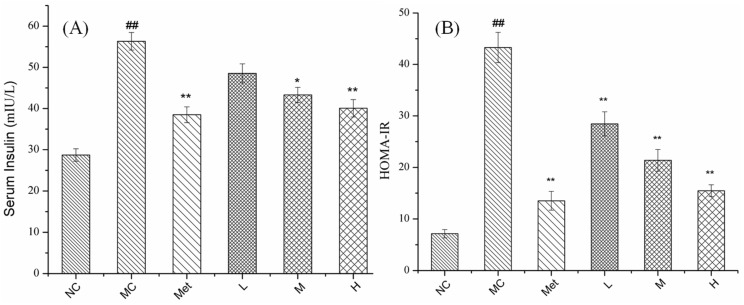
Effects of PPE on insulin and insulin resistance index in mice. (**A**) serum insulin; (**B**) insulin resistance index (HOMA-IR). NC, normal control group; MC, model control group; Met, positive control group; L, M, and H groups administrated with 50, 100, and 150 mg PPE/kg/bw, respectively. ^##^ *p* < 0.01 vs. NC, * *p* < 0.05, ** *p* < 0.01 vs. MC.

IR is a pre-diabetic characteristic that is generally considered to be a decreased sensitivity to insulin response [26]. Excessive inflammatory factors in adipocytes induce serine 307 phosphorylation on insulin receptor substrate 1 (IRS-1) and block tyrosine phosphorylation, thus impair the insulin signaling pathway and induce insulin resistance [30]. As shown in Figure 5A, the serum insulin content in the MC group was significantly higher than that in the NC group (*p* < 0.01), and metformin could significantly reduce the serum insulin content of the T2DM mice (*p* < 0.01). When the T2DM mice were treated with PPE, the serum insulin level decreased and was positively correlated with the PPE concentration. The serum insulin level in the MC group was significantly decreased from 56.31 ± 2.13 mIU/L to 40.05 ± 2.11 mIU/L after a high-dose of 150 mg/kg/bw of PPE administration (*p* < 0.01). Moreover, the IR index of STZ-induced diabetic mice was significantly elevated compaerd with the NC group (*p* < 0.01), indicating that the model mice appeared insulin resistance (Figure 5B). Both metformin and PPE could significantly reduce the insulin resistance index of diabetic mice and improve the state of insulin resistance (*p* < 0.01).

### 3.6. Effects of PPE on Serum Glucose Metabolism in Mice

As a global metabolic disease, T2DM is characterized by persistent hyperglycemia [29]. Glycosylated hemoglobin is an important tool for the diagnosis and management of diabetes, and is known as the gold standard for blood glucose control. As a typical stress hormone, epinephrine can induce metabolic syndrome such as short-term systolic hypertension and hyperglycemia [31].

**Figure 6:**
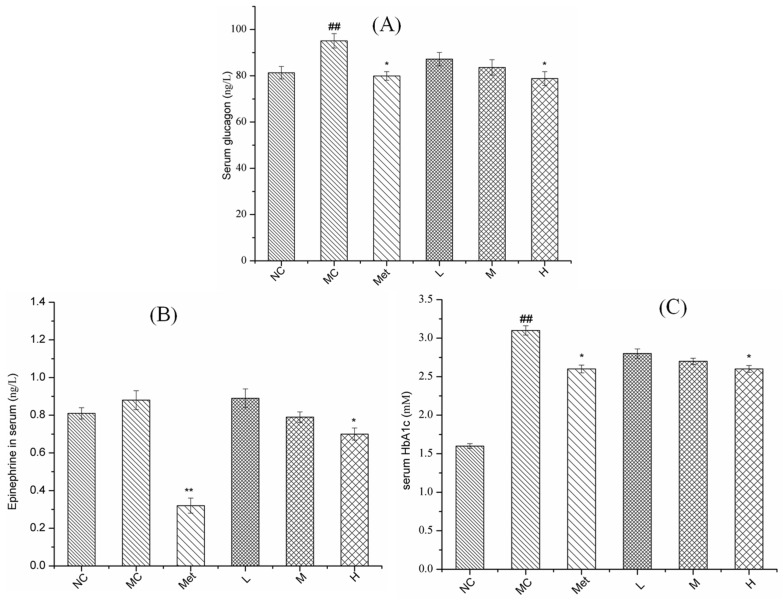
Effect of PPE on serum glucose metabolism in mice. The contents of glucagon (**A**), epinephrine (**B**) and glycosylated hemoglobin (**C**) in serum. NC, normal control group; MC, model control group; Met, positive control group; L, M, and H groups administrated with 50, 100, and 150 mg PPE/kg/bw, respectively. ^##^ *p* < 0.01 vs. NC, * *p* < 0.05, ** *p* < 0.01 vs. MC.

As observed from Figure 6, the contents of glucagon, epinephrine and glycosylated hemoglobin in the MC group were significantly increased compared with in NC group (*p* < 0.01). After the low and medium dose PPE treatment, all three indicators of serum glucose metabolism demonstrated a downward trend without significant difference (*p* > 0.05). When diabetic mice were treated with metformin and high-dose PPE, all the contents of glucagon, epinephrine and glycosylated hemoglobin were significantly reduced (*p* < 0.01). Glucagon can increase the hepatic adipose triglyceride lipase activity, intrahepatic lipopolysis and hepatic acetyl-CoA content, and then stimulates hepatic gluconeogenesis, while it also increases mitochondrial fat oxidation [32]. Epinephrine is considered a master regulator of lipolysis [33]. The study found that mice that could not produce epinephrine had normal metabolism on a normal 14% fat diet, but when fed a high-fat diet, they developed hyperglycemic and IR [31]. Thus, PPE reduced glucagon and might contribute to T2DM management via improvement of lipid metabolism.

### 3.7. Effects of PPE on Serum Lipid Metabolism in Mice

**Figure 7:**
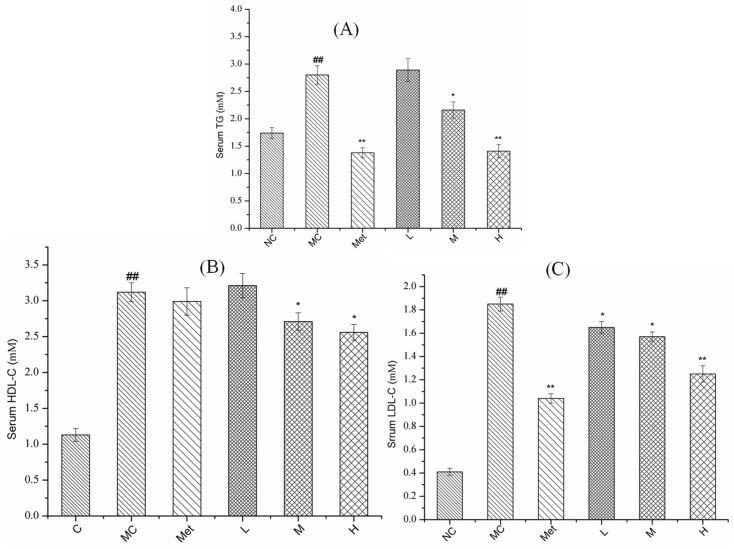
Effect of PPE on serum lipid metabolism in mice. The contents of TG (**A**), HDL-C (**B**) and LDL-C (**C**) in serum. NC, normal control group; MC, model control group; Met, positive control group; L, M, and H groups administrated with 50, 100, and 150 mg PPE/kg/bw, respectively. ^##^ *p* < 0.01 vs. NC, * *p* < 0.05, ** *p* < 0.01 vs. MC.

The determination of serum TG, HDL-C, and LDL-C could reflect the level of lipid metabolism, as presented in Figure 7.

The serum TG, HDL-C and LDL-C level in MC group were significantly increased compared with in the NC group (*p* < 0.01). Metformin significantly reduced the serum TG and LDL-C levels of the T2DM mice (*p* < 0.01). When the T2DM mice were treated with PPE, the levels ofserum TG, HDL-C, and LDL-C decreased in a dose-dependent manner. In addition, the level of LDL-C in serum of the T2DM mice were significantly decreased by gavage with low dose of PPE compared with the MC group (*p* < 0.05). Furthermore, compared with the MC group, the TG, HDL-C and LDL-C levels in the serum of diabetic mice were significantly reduced by the medium and high dose of PPE treatment (*p* < 0.05). In accordance with our prediction, PPE significantly reduced lipid levels in T2DM model mice.

### 3.8. Effects of PPE on Gene Expression in the IRS1/PI3K/AKT Pathway in Mouse Liver

**Figure 8:**
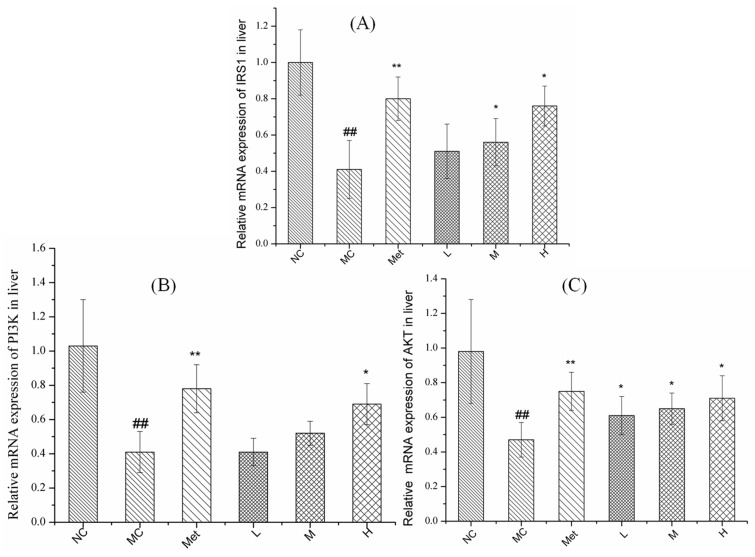
Expression of IRS1 (**A**), PI3K (**B**) and AKT (**C**) mRNA in mice. NC, normal control group; MC, model control group; Met, positive control group; L, M, and H groups administrated with 50, 100, and 150 mg PPE/kg/bw, respectively. ^##^ *p* < 0.01 vs. NC, * *p* < 0.05, ** *p* < 0.01 vs. MC.

The IRS1/PI3K/AKT signalling pathway is associated with the formation of T2DM [34]. As shown in Figure 8, the hepatic IRS1, PI3K and AKT mRNA expression in the MC group were significantly reduced compared with those in the NC group (*p* < 0.01). Compared to the T2DM mice, the hepatic mRNA expression of AKT was significantly increased in the low-dose group (*p* < 0.05), mRNA expression of IRS1 and AKT was significantly enhanced in the medium-dose group (*p* < 0.05), and mRNA expression of IRS1, PI3K and AKT were significantly improved in the high-dose PPE group and metformin group (*p* < 0.05).

These data suggest that PPE may play an anti-diabetic role by activating the hepatic mRNA expression level of IRS1/PI3K/AKT signalling pathway in T2DM mice. These results were in accordance with the previous studies that ethanol and ethanol extracts of *P. baumii* fruiting body revealed obviously hypoglycemic effect on STZ-induced diabetic mice [11]. In summary, we propose that PPE are the active components in *P. baumii*, accountable for reducing serum glucose presumably through the IRS1/PI3K/AKT pathway.

### 3.9. Effects of PPE on the Histomorphology of Mouse Liver

**Figure 9:**
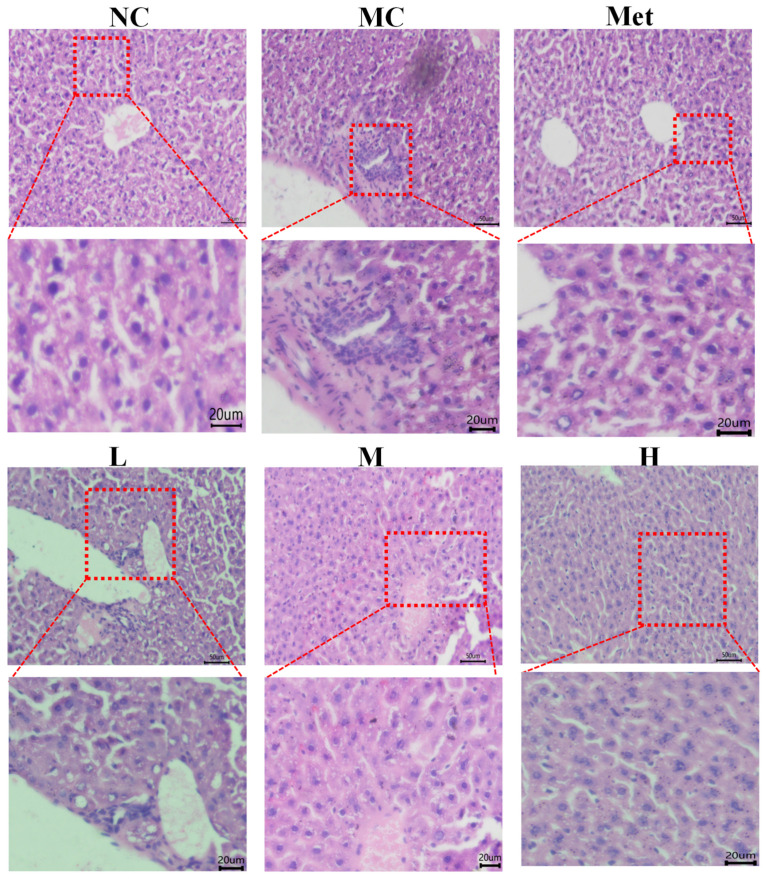
Hematoxylin & eosin staining and morphology for liver tissue. NC, normal control group; MC, model control group; Met, positive control group; L, M, and H groups administrated with 50, 100, and 150 mg PPE/kg/bw, respectively.

The pathological morphology of liver tissues in mice is shown in Figure 9. In the normal control group (A), the morphology of liver tissues is normal and regular, the structure of liver lobules is clear, the liver sinuses are normal, and the structure of nuclei is clear. The hepatocytes of model mice (B) induced by STZ were disordered, accompanied by a large amount of lipid droplets and rich fat content. STZ-induced diabetes is generally accompanied by abnormal liver function, which can exacerbate the severity of naturally occurring diabetes [35]. In addition, persistent or chronic hyperglycemia can cause serious microvascular complications and consequent organ damage [36]. After treatment with PPE and metformin, the number of lipid droplets in liver cells decreased significantly. PPE treatment could effectively reduce liver injury and improve abnormal serum biochemical indexes, which may be related to the antioxidant and anti-inflammatory effects of PPE basing on our previous and presented work [14].

## 4. Conclusions

The study is intended to explore the anti-inflammatory properties and in vivo antihyperglycemic activity of PPE and the associated underlying mechanisms, on the basis of our previous work in vitro [14]. The results showed that the supplementation of PPE by weights in the diet could dose-dependently reduce plasma TG, LDL-C and HDL-C, respectively. T2DM is characterized by higher FBG levels, lower glucose tolerance, and symptoms such as IR and dyslipidemia, etc. [37]. As expected, in the present study, FBG levels and OGTT were significantly increased in T2DM mice compared with the control mice, while intervention by PPE significantly reversed the upregulation of FBG and OGTT. The liver is one of the important organs in glucose metabolism and also one of the important target organs of insulin. Insulin combined with hepatic insulin receptors on the cell membrane, bound to the subunit P85, and regulated PI3K. Activated PI3K, second messenger PPI3, and activation of downstream of the insulin receptors can promote AKT phosphorylation [38]. The activation of AKT promotes glucose transporter vesicles to the cell membrane, thereby eliciting the transport of extracellular glucose into the cell [39], and also reduces gluconeogenesis. PPE can promote the phosphorylation of IRS1 in the liver of diabetic mice, and increase the PI3K mRNA expression. The specific effect can be reflected in some biochemical indicators and liver status. Moreover, the results of this study suggest that PPE reduced blood glucose levels, improved IR and glycogen storage, and thus improved glucose metabolism abnormalities in T2DM mouse models. The mechanism of action may be that PPE reduces inflammation and relieves IR by the activation of the IRS-1/PI3K/AKT pathway. Thus, our findings imply that PPE can be a new food ingredient for treating T2DM. The results suggest that the phenolics in the cultivated *P. baumii* fruit body exibit anti-inflammatory properties and improve symptoms of diabetes.

Taken together, this study fills a gap in the anti-diabetic effects of the phenolics from cultivated *P. baumii* in vivo. However, further in vitro and in vivo in-depth studies are worth to clarify the potential mechanism of its regulation of T2DM. For example, the utilization of polyphenols in the intestinal tract is very low. The hypoglycemic effect of PPE may be to regulate intestinal microbiota and play a role through the enterohepatic axis, so it is necessary to carry out future studies in this field. In short PPE treatment has a certain improvement effect on STZ-induced glucose metabolic disorders, and the condition of T2DM is significantly improved.

## Figures and Tables

**Table 1:** Effect of PPE on body weight of mice

Group	Body Weight of Mice (g)						
	0 d	10 d	20 d	30 d	40 d	50 d	60 d
NC	25.2 ± 1.1	26.4 ± 1.0	28.7 ± 1.1	31.1 ± 1.5	33.0 ± 1.5	34.9 ± 1.2	35.9 ± 1.5
MC	28.2 ± 1.0 ^##^	29.7 ± 1.2 ^##^	32.4 ± 1.3 ^##^	35.0 ± 1.4 ^##^	38.3 ± 1.5 ^##^	40.4 ± 1.6 ^##^	42.1 ± 2.2
Met	28.4 ± 1.1	29.6 ± 1.2	32.3 ± 1.6	34.1 ± 1.7	36.2 ± 1.7 *	38.4 ± 1.9 *	39.8 ± 2.3 *
L	27.7 ± 1.6	28.7 ± 1.7	31.5 ± 1.8	33.6 ± 1.9	36.1 ± 2.2 **	38.7 ± 2.7 *	40.2 ± 3.1
M	28.3 ± 1.5	29.4 ± 1.8	32.4 ± 2.1	34.7 ± 2.2	36.8 ± 2.2	38.9 ± 2.1	40.4 ± 2.2
H	27.9 ± 1.5	28.9 ± 1.5	31.7 ± 2.0	33.9 ± 1.9	36.1 ± 2.0 **	38.2 ± 2.1 *	39.3 ± 2.1 **
**Group**	**Body Weight Gain of Mice (g)**						
NC	0	1.2 ± 0.6	3.5 ± 1.3	5.9 ± 1.8	7.8 ± 1.7	9.6 ± 1.54	10.7 ± 1.6
MC	0	1.5 ± 0.4	4.3 ± 0.8	6.8 ± 0.7	10.1 ± 0.8 ^##^	12.3 ± 1.1 ^##^	13.9 ± 1.6 ^##^
Met	0	1.2 ± 0.3	3.9 ± 0.8	5.7 ± 0.8 *	7.7 ± 0.9 **	10.0 ± 1.3 **	11.4 ± 1.6 **
L	0	0.9 ± 0.3 **	3.7 ± 0.8	5.9 ± 0.8	8.3 ± 1.1 **	11.0 ± 1.58 *	12.5 ± 1.9 *
M	0	1.1 ± 0.5 *	4.0 ± 0.7	6.4 ± 1.0	8.5 ± 1.0 **	10.5 ± 1.3 **	12.0 ± 1.1 **
H	0	1.0 ± 0.6 *	3.7 ± 1.0	6.0 ± 1.4	8.1 ± 1.7 **	10.2 ± 1.9 **	11.4 ± 2.1 **

NC, normal control group; MC, model control group; Met, positive control group; L, M, and H groups administrated with 50, 100, and 150 mg PPE/kg/bw, respectively. ^##^ *p <* 0.01 vs. NC; * *p* < 0.05, ** *p <* 0.01 vs. MC.

## Data Availability

The data presented in this study are available on request from the corresponding author.

## Supplementary Materials

The following are available online. Table S1: Primer sequences for Q-PCR.

## Sample Availability

Samples of the compounds are not available from the authors.
