# Supplementary material for: Anti-Inflammatory Properties In Vitro and Hypoglycaemic Effects of Phenolics from Cultivated Fruit Body of Phellinus baumii in Type 2 Diabetic Mice
Source: Molecules. 2021 Apr 15;26(8):2285. doi: 10.3390/molecules26082285 (PMC8071318; doi:10.3390/molecules26082285)
Supplement: Supplementary file 1 [file molecules-26-02285-s001.zip › molecules-1137664-supplementary.pdf]

table S1. Primer sequences for Q-PCR

Target	Forward primers (5'-3')	Reverse primers (5'-3')	Product length (bp)
PI3K	ACAAAGCTCTACTCTAGGCGTG	TTACCAGCATGGTCATGGGC	144
AKT	AGAGAGCCGAGTCCTACAGAATA	CCGAGAGAGGTGGAAAAACA	133
IRS-1	TGTGCCAAGCAACAAGAAAG	ACGGTTTCAGAGCAGAGGAA	171
β-actin	GATCGATGCCGGTGCTAAGA	TCCTATGGGAGAACGGCAGA	137
